# A Clinical Decision Support System for Diabetes Patients with Deep Learning: Experience of a Taiwan Medical Center

**DOI:** 10.7150/ijms.71341

**Published:** 2022-06-13

**Authors:** Ting-Ying Chien, Hsien-Wei Ting, Chih-Fang Chen, Cheng-Zen Yang, Chong-Yi Chen

**Affiliations:** 1 Department of Computer Science and Engineering, Yuan Ze University, Taoyuan City, Taiwan.; 2 Innovation Center for Big Data and Digital Convergence, Yuan Ze University, Taoyuan City, Taiwan.; 3 Graduated Program in Biomedical Informatics, Yuan Ze University, Taoyuan City, Taiwan.; 4 Department of Neurosurgery, Taipei Hospital, Ministry of Health and Welfare, New Taipei City, Taiwan.; 5 Department of Pharmacy, MacKay Memorial Hospital, Taipei City, Taiwan.

**Keywords:** Bidirectional Long Short-Term Memory (Bi-LSTM), Clinical Decision Support System (CDSS), Deep Learning, Diabetes Mellitus, Electronic Health Record, Oral Hypoglycemic Agents

## Abstract

**Background:** Diabetes mellitus (DM) is a major public health problem worldwide. It involves dysfunction of blood sugar regulation resulting from insulin resistance, inadequate insulin secretion, or excessive glucagon secretion.

**Methods:** This study collated 971,401 drug usage records of 51,009 DM patients. These data include patient identification code, age, gender, outpatient visiting dates, visiting code, medication features (included items, doses, and frequencies of drugs), HbA1c results, and testing time. We apply a random forest (RF) model for feature selection and implement a regression model with the bidirectional long short-term memory (Bi-LSTM) deep learning architecture. Finally, we use the root mean square error (RMSE) as the evaluation index for the prediction model.

**Results:** After data cleaning, the data included 8,729 male and 9,115 female cases. Metformin was the most important feature suggested by the RF model, followed by glimepiride, acarbose, pioglitazone, glibenclamide, gliclazide, repaglinide, nateglinide, sitagliptin, and vildagliptin. The model performed better with the past two seasons in the training data than with additional seasons. Further, the Bi-LSTM architecture model performed better than support vector machines (SVMs).

**Discussion & Conclusion:** This study found that Bi-LSTM models is a well kernel in a CDSS which help physicians' decision-making, and the increasing the number of seasons will negative impact the performance. In addition, this study found that the most important drug is metformin, which is recommended as first-line treatment OHA in various situations for DM patients.

## Introduction

Diabetes mellitus (DM) is a major public health problem worldwide. It involves dysfunction of blood sugar regulation resulting from insulin resistance, inadequate insulin secretion, or excessive glucagon secretion 1. There are two types of DM. Type 1 DM is usually due to an autoimmune disorder and involves the destruction of pancreatic beta-cells. Type 2 DM is caused by impairment of glucose regulation due to the malfunction of pancreatic beta cells or insulin resistance 1. Treatment using oral hypoglycemic agents (OHA) for type 2 DM may have negative side effects, such as hypoglycemia. Therefore, it is crucial to ensure the safety and efficacy of OHA usage 1-7.

Clinical decision support systems (CDSSs), which integrate electronic health records (EHRs) and expert knowledge, have improved the decision-making of physicians and medical caregivers 8-12. Many methods have been developed for CDSSs, including linear/logistic regression, support vector machines (SVMs), decision trees, random forest (RF), rough sets, and trajectory methods 13-19. Glycated hemoglobin (HbA1c) is extensively studied in these approaches because it is a good indicator of DM control. DM patients with higher Hba1c measures are more likely to experience renal diseases, macrovascular events, cardiovascular diseases, retinopathies, skin ulceration/gangrene, and high mortality 3. A well-controlled HbA1c value plays an important role in DM management. CDSSs have been shown to be effective in supporting HbA1c control. For example, O'Connor *et al*. showed that the HbA1c of DM patients significantly improve when the physicians use a CDSS compared with when physicians do not use a CDSS (*p* < 0.01). Moreover, 94% of physicians using the CDSS were satisfied for this application and physicians continued to use the CDSS for more than one year without research funding support 20.

**Figure 1:**
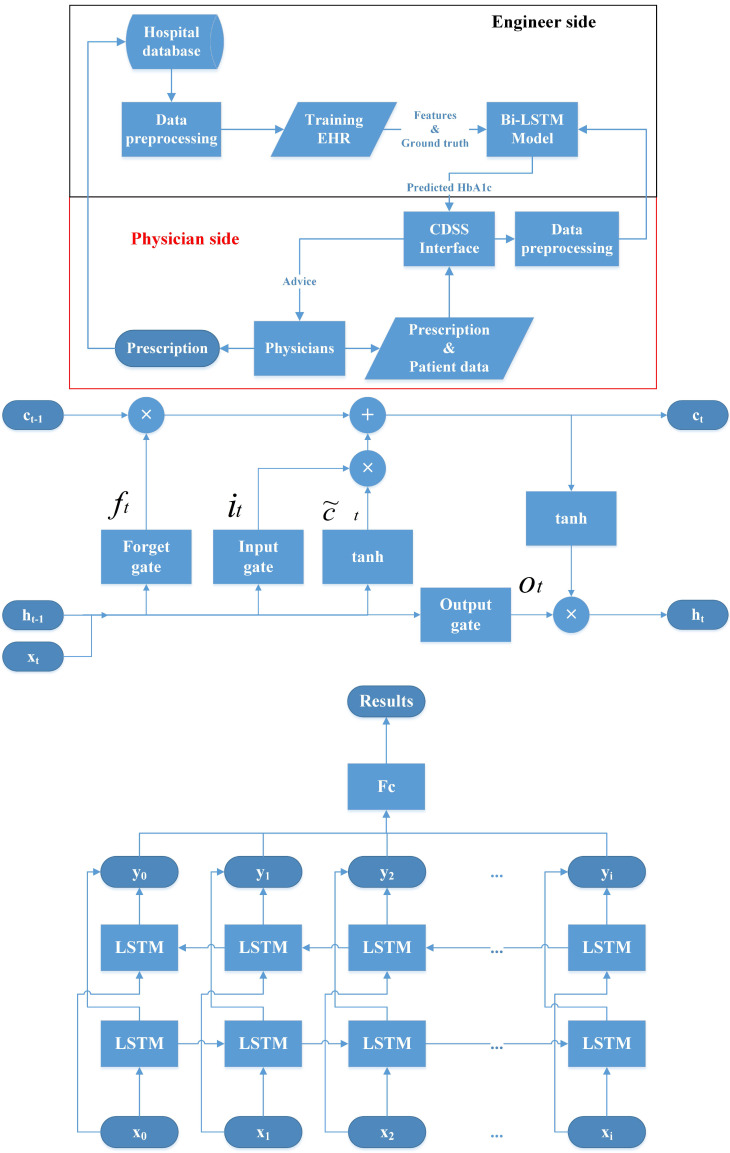
Structure of the clinical decision support system (CDSS), Long short time model (LSTM) and bidirectional-LSTM (Bi-LSTM) model. CDSS: clinical decision support system; EHR: electronic health records; Bi-LSTM: Bidirectional long short-term memory network model.

Recently, deep learning methods have dramatically improved different fields of medical care and research 21, 22. They have also been used as the core methods to build the CDSS 23, 24. For example, convolutional neural networks (CNNs) are used to process image data and recurrent neural networks (RNNs) are used for sequential pattern problems 23, 25. Sun *et al.* proposed a method to predict blood sugar levels at four intervals, namely 15, 30, 45, and 60 minutes, using the long short-term memory (LSTM) model and the bidirectional-LSTM (Bi-LSTM) model 26. Therefore, we devise a CDSS using a Bi-LSTM model with HbA1c as the outcome index for managing OHA usage. The structure of the proposed CDSS, the LSTM model, and the Bi-LSTM model are shown in Figure 1.

## Materials and Methods

**Figure 2:**
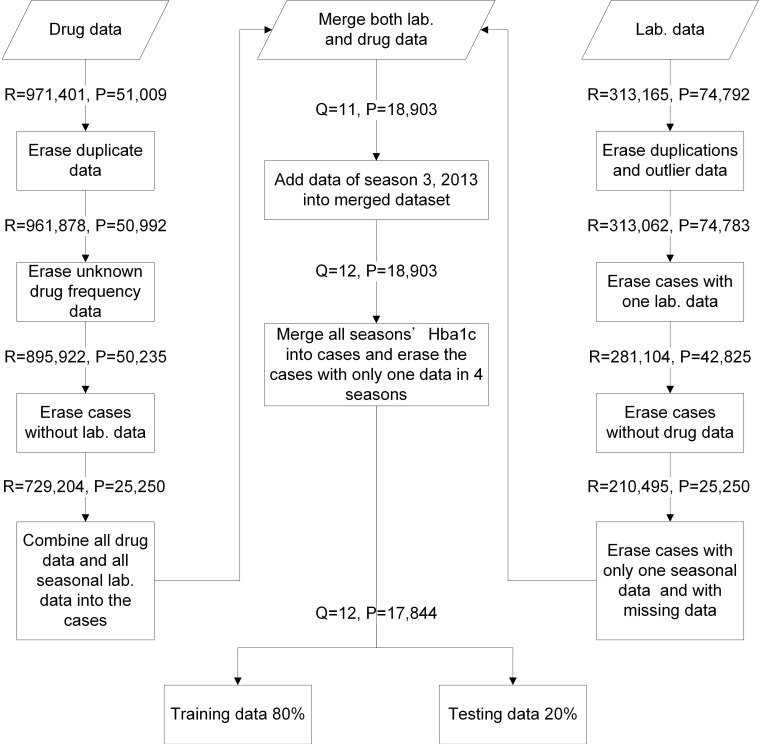
Data management flowchart. R: visiting times; P: patient case numbers; Q: seasonal times; Lab. data: laboratory data.

We collated 971,401 drug usage records of 51,009 diabetes mellitus (DM) patients from January 2012 to December 2014 (12 seasons) and 313,165 laboratory records of 74,792 DM patients in a medical center from January 2012 to June 2015 (14 seasons). These data included patient identification code, age, gender, outpatient visiting dates, visiting code, medication features (included items, doses, and frequencies of drugs), HbA1c results, and testing time. The data were combined and cleansed. Twelve seasons of data and 17,844 DM patients were included in this study. The data were evaluated with five-fold cross-validation (training data = 80% and testing data = 20%) (Figure 2). We applied an RF model with mean square error (MSE) for feature selection where higher mean decrease MSE indicated more important parameters 27-29. OHA dosages and codes were collected. This study was approved by the Institutional Review Board (IRB) of the MacKay Memorial Hospital (IRB approval number: 15MMHIS143e).

We implemented the Bi-LSTM structure using PyTorch running on two personal computers equipped, respectively, with Ubuntu 16.04 and Ubuntu 18.04. The GPU environments were NVIDIA GeForce GTX 980 and GTX 1080 Ti. We used Grid search to adjust the model parameters: epoch (100); batch size (32, 64, 128); hidden layer neuron numbers of Bi-LSTM (32, 64, 128); dropout rate (0, 0.2) between Bi-LSTM; optimizers AMSBound 30 and Adadelda 31; and learning rate (AMSBound used 0.001 and the Adadelda used 1.0). We used early stopping (patience = 30) and L2 normalization (weight decay = 0.0005) to solve the problem of overfitting and applied gradient clipping (clip norm = 5) to prevent exploding gradient problems. We also implemented a support vector regression (SVR) model using Rgtsvm in R to compare with the Bi-LSTM model 18.

To evaluate the models, we used root mean square error (RMSE) (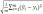), sensitivity (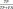), specificity (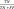), and the Matthews correlation coefficient (MCC) (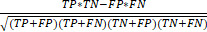). We applied Pearson's chi-squared test and the student t-test for data analysis. The statistical analysis was conducted using SPSS version 19.0 (SPSS Inc., Chicago, IL, USA). Statistical significance was defined as *p* < 0.05.

## Results

Of the included 17,844 cases, 8729 (49.0%) were male and 9115 (51.0%) were female. The mean age was 62.3 years old (SD = 11.9) overall, 60.4 (SD = 11.8) for males, and 64.2 (11.7) for females. The 45 to 64 year old age had the most cases (8,507 cases), followed by those aged above 65(6,966 cases). The mean Hba1c was 7.6% (SD = 1.7). There were 13,346 cases with Hba1c higher than 6.5% and 4,498 cases whose Hba1c were less than 6.5% (Table 1).

The data included 11 types of drugs. Compound drugs glimepiride (25,719), pioglitazone (25,720), and vildagliptin (25,726) were combined with metformin dosages of 500, 850, and 1000 mg, respectively. Nateglinide had dosages of 60 and 120 mg.

The most important feature was metformin with mean decreased MSE = 171.8, followed by glimepiride (156.6), acarbose (151.8), pioglitazone (148.1), glibenclamide (143.7), gliclazide (114,1), repaglinide (93.3), nateglinide (80.6), sitagliptin (74.0), and vildagliptin (21.9) (Table 2).

This study treated every season as ground truth from 2013 Q1 to 2015 Q1 and constructed nine datasets, each having a different sample size. For example, the dataset of 2014 Q4 had 12,677 and 3169 cases as training and test samples, respectively. Using other data as independent factors, we designed three kinds of models. The first used two seasons of data to predict drug usage of the third season. For example, model 9 (2015 Q1) used 2014 Q3 and 2014 Q4 to predict 2015 Q1. The other two types of model used three/four seasons to predict the drugs of the fourth/fifth seasons.

This study also evaluated differences in Hba1c between seasons. For example, we calculated the differences in mean Hba1c between 2015 Q1 and 2014 Q4 (0.87%), 2014 Q3 (0.98%) and 2014 Q2 (1.09%). We found that longer time distances had greater differences in Hba1c (Table 3).

We compared Bi-LSTM and SVM in the two-, three-, and four-season models. The RMSE of both two-season models (Bi-LSTM = 1.05±0.17 and. SVM = 1.05±0.07) was the best, followed by the three-season models (Bi-LSTM = 1.10±0.25 and. SVM = 1.12±0.03) and four-season models (Bi-LSTM = 1.09±0.21 and SVM = 1.16±0.04).

The sensitivity of the SVM models were not significantly different to each other (two seasons: 0.88±0.03, three seasons: 0.88±0.02, four seasons: 0.89±0.02). The sensitivity of the Bi-LSTM models gradually decreased non-significantly (two seasons: 0.83±0.16, three seasons: 0.80±0.21, four seasons: 0.77±0.23), but performed worse than the SVM models.

The specificity of the SVM models gradually decreased as the included seasons increased (two seasons: 0.68±0.05, three seasons: 0.64±0.05, four seasons: 0.59±0.04). The specificity of the Bi-LSTM models was not significant for any approach (two seasons: 0.69±0.32, three seasons: 0.71±0.31, four seasons: 0.71±0.30). According to the MCC evaluation, there were no significant differences between the six models. The two-season SVM model (0.39±0.06) had the shortest run time, followed by the three-season (0.47±0.09) and four-season (0.52±0.06) SVM models. The Bi-LSTM models had significantly longer run times (two seasons: 3.39±0.64, three seasons: 5.18±1.07, four seasons: 5.36±1.17) than the SVM models (Table 4).

## Discussion & Conclusion

Studies have found that higher Hba1c is linked with increased risk of complications in DM patients 3. A physician-pharmacist collaboration is useful for OHA adjustment to manage Hba1c owing to physician knowledge and experience 32. However, it is a challenge to leverage the knowledge of these experts. The current study found that Bi-LSTM models performed better than SVM models for a CDSS to support physicians' decision-making related to OHA adjustment for DM patients. Many CDSS and classification models have used SVM and other artificial intelligence technologies 18, 33-36.

We also found that increasing the number of seasons used in the prediction negatively impacted accuracy and RMSE. Physicians reference the most recent Hba1c value to adjust OHA dosage and may choose to maintain the dosage if the Hba1c value is only slightly higher than 7% for the first time. Thus, it is not necessary to reference three or more seasons of Hba1c data, as validated by our experimental results.

We calculated the importance of these drugs and used RF to translate this information into the CDSS 27-29, 33, 35. The most important drug was metformin, which is recommended as first-line treatment OHA in various situations for DM patients. This drug improves lipids and inflammatory markers and reduces cardiovascular events, but may be contraindicated for patients with mild to moderate chronic kidney disease. Recent research indicates that metformin requires caution in these kinds of DM patients 5. Sulfonylureas is an important DM drug. We found that the glimepiride (2^nd^) glibenclamide (5^th^), and gliclazide (6^th^) are also important OHA for DM patients 2, 4. Acarbose was also found to have some gastrointestinal adverse effects, which were similar to metformin 6. Pioglitazone is an important DM drug that may reduce Hba1c and improve both metabolic syndrome and nonalcoholic fatty liver disease/nonalcoholic steatohepatitis 7. It also has a side effect of weight loss for some patients, which is sometimes treated as a benefit. Glimepiride, pioglitazone, and vildagliptin were all combined with metformin (Table 2).

This study has some limitations and areas for extension. Yanase et al. reported that low HbA1c is linked with frailty and suspected malnutrition in elderly type 2 DM patients 37. Around 25% of cases in this study had Hba1c < 6.5%, indicating that the DM control was too strict for some patients, potentially leading to malnutrition or hypoglycemia. Our CDSS defined “good control” as Hba1c ≤ 7. Although our approach worked well, future versions could be enhanced by considering 6.5 ≤ Hba1c ≤ 7.

## Figures and Tables

**Table 1:** Demographic data of training cases

Sex	Male	Female	All
Case number (%)	8,729 (49.0)	9,115 (51.0)	17,844
Mean age (SD)	60.4 (11.8)	64.2 (11.7)	62.3 (11.9)
**Age rank**			
<25 years old	22	20	42
25-44 years old	732	373	1,105
45-64 years old	4,531	3,976	8,507
≥ 65 years old	2,882	4,084	6,966
Unknown age	562	662	1,224
Mean Hba1c (SD)	7.6% (1.8)	7.6% (1.7)	7.6% (1.7)
Hba1c ≤ 6.5% (%)	2,337 (26.8%)	2,161 (23.7%)	4,498 (25.2%)
Hba1c > 6.5% (%)	6,392 (73,2%)	6,954 (76,3%)	13,346 (74.8%)

**Table 2:** Oral hypoglycemic agents (OHA) mean decreased mean square error (MSE), dosages, and codes

OHA	₸ Mean Decrease MSE					Dosage
	All	Male	Female	≥65 y/o	<65 y/o	
Metformin	171.8	108.69	117.61	94.56	114.81	500mg
^#^Glimepiride	156.6	77.13	100.00	89.72	72.88	2mg
Acarbose	151.8	98.81	55.44	80.20	49.18	100mg
^#^Pioglitazone	148.1	24.31	29.57	12.14	26.65	15mg
Glibenclamide	143.7	33.78	73.28	52.01	63.91	5mg
Gliclazide	114.1	97.96	94.60	96.95	82.94	30mg
Repaglinide	93.3	42.00	50.40	37.41	51.21	1mg
^§^Nateglinide	80.6	8.75	20.65	19.39	23.10	60mg, 120mg
Sitagliptin	74.0	55.33	76.43	91.87	45.17	100mg
^#^Vildagliptin	71.1	44.95	35.64	44.73	42.04	50mg
Linagliptin	21.9	8.92	4.85	-0.38	2.75	5mg

# These three compound drugs are all combined with metformin. Glimepiride, pioglitazone, and vildagliptin have 500, 850, and 1000 mg of metformin added, respectively. § Nateglinide has two dosages: 60 and 120 mg. ₸ MSE: mean square error.

**Table 3:** Research design and Hba1c differences between first and last seasons in each model. There are nine datasets. The models use two/three/four seasons to predict the drugs for the third/fourth/fifth seasons.

Dataset	Ground truth	Two seasons		Three seasons		Four seasons		Training sample	Testing sample
		Time period	HBa1 difference	Time period	HBa1 difference	Time period	HBa1 difference		
9	2015 Q1	2014 Q3 -2014 Q4	0.87	2014 Q2 -2014 Q4	0.98	2014 Q1 -2014 Q4	1.09	12334	3084
8	2014 Q4	2014 Q2 -2014 Q3	0.88	2014 Q1 -2014 Q3	1.02	2013 Q4 -2014 Q3	1.10	12677	3169
7	2014 Q3	2014 Q1 -2014 Q2	0.92	2013 Q4 -2014 Q2	1.02	2013 Q3 -2014 Q2	1.14	8626	2156
6	2014 Q2	2013 Q4 -2014 Q1	0.90	2013 Q3 -2014 Q1	1.07	2013 Q2 -2014 Q1	1.23	12362	3090
5	2014 Q1	2013 Q3 -2013 Q4	0.99	2013 Q2 -2013 Q4	1.17	2013 Q1 -2013 Q4	1.25	12474	3119
4	2013 Q4	2013 Q2 -2013 Q3	1.08	2013 Q1 -2013 Q3	1.17	2012 Q4 -2013 Q3	1.30	12266	3066
3	2013 Q3	2013 Q1 -2013 Q2	1.02	2012 Q4 -2013 Q2	1.18	2012 Q3 -2013 Q2	1.30	11826	2957
2	2013 Q2	2012 Q4 -2013 Q1	1.03	2012 Q3 -2013 Q1	1.19	2012 Q2 -2013 Q1	1.30	11442	2861
1	2013 Q1	2012 Q3 -2012 Q4	1.03	2012 Q2 -2012 Q4	1.18	2012 Q1 -2012 Q4	1.31	8155	2039

**Table 4:**
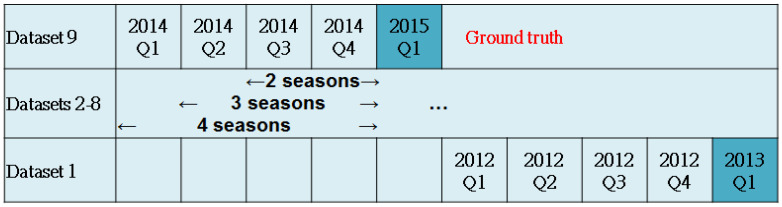
Comparison of Bi-LSTM and SVM models with different seasons

Seasonal model	Evaluation index	SVM	Bi-LSTM
Two seasons	RMSE	1.05±0.07	1.05±0.17
	Sensitivity	0.88±0.03	0.83±0.16
	Specificity	0.68±0.05	0.69±0.32
	MCC	0.57±0.05	0.56±0.15
	Duration (Hours)	0.39±0.06	3.39±0.64
Three seasons	RMSE	1.12±0.03	1.10±0.25
	Sensitivity	0.88±0.02	0.80±0.21
	specificity	0.64±0.05	0.71±0.31
	MCC	0.54±0.05	0.55±0.15
	Duration (Hours)	0.47±0.09	5.18±1.07
Four seasons	RMSE	1.16±0.04	1.09±0.21
	sensitivity	0.89±0.02	0.77±0.23
	specificity	0.59±0.04	0.71±0.30
	MCC	0.50±0.04	0.54±0.12
	Duration (Hours)	0.52±0.06	5.36±1.17

Bi-LSTM: Bidirectional long short-term model. SVM: support vector machine. Root mean square error (RMSE) =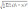; Sensitivity = 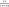; Specificity = 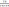; Matthews correlation coefficient (MCC) = 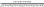.
